# Diverse Applications of the Anti-Diabetic Drug Metformin in Treating Human Disease

**DOI:** 10.3390/ph17121601

**Published:** 2024-11-27

**Authors:** Chris-Tiann Roberts, Nicole Raabe, Lara Wiegand, Ashraf Kadar Shahib, Mojgan Rastegar

**Affiliations:** Department of Biochemistry and Medical Genetics, Max Rady College of Medicine, Rady Faculty of Health Sciences, University of Manitoba, Winnipeg, MB R3E 0J9, Canada

**Keywords:** metformin, drug repurposing, epigenetics, human disease, type 2 diabetes

## Abstract

Metformin is a commonly used drug for treating type 2 diabetes. Metformin is an inexpensive drug with low/no side effects and is well tolerated in human patients of different ages. Recent therapeutic strategies for human disease have considered the benefits of drug repurposing. This includes the use of the anti-diabetic drug metformin. Accordingly, the anti-inflammatory, anti-cancer, anti-viral, neuroprotective, and cardioprotective potentials of metformin have deemed it a suitable candidate for treating a plethora of human diseases. As results from preclinical studies using cellular and animal model systems appear promising, clinical trials with metformin in the context of non-diabetes-related illnesses have been started. Here, we aim to provide a comprehensive overview of the therapeutic potential of metformin in different animal models of human disease and its suggested relationship to epigenetics and ailments with epigenetic components.

## 1. Introduction

Originally, drug repurposing (also known as drug repositioning, redirecting, or reprofiling) was described as a process of discovering new applications for drugs outside of the realm for which they were originally developed [1]. Today, the concept of drug repurposing has broadened to include active substances that have failed clinical phase development and drugs withdrawn from the market [1]. Ideally, any drug that is a candidate for repurposing has undergone clinical drug development as well as safety and toxicity studies. However, drug repurposing may also occur during clinical trials. A notable example is the drug repurposing of Viagra (Sildenafil) [2]. Originally, Sildenafil was explored for the treatment of angina pectoris *due to* its potential and selectivity in inhibiting phosphodiesterase 5 (PDE5) [2]. The drug proved less promising as a therapeutic agent for angina. During clinical trials, however, PDE5 inhibition was recognized as a novel approach to erectile dysfunction (ED). After 21 separate clinical trials, Viagra was approved for the treatment of ED. In years to follow, Viagra was also approved by the U.S. Food and Drug Administration (U.S. FDA, Silver Spring, MD, USA) for the treatment of pulmonary arterial hypertension [3]. The vitality of repurposed drugs has become even more evident during the COVID-19 pandemic, where drugs from diverse classes were explored for COVID-19 treatment [4]. These examples, among others, underscore the multifaceted nature of drugs and their application to conditions outside of the scope of their intended purpose.

Like other drugs, the therapeutic potential of the anti-diabetic drug metformin extends beyond its initial intended use. Currently, it is estimated that metformin is prescribed to over 150 million people worldwide and is considered an essential drug, as recorded on the World Health Organization Model List of Essential Medicines [5]. Despite a lack of consensus on the exact mechanism(s) by which metformin acts, its pleiotropic action on biochemical pathways has piqued scientific curiosity. As a result, there is a rapidly expanding body of literature surrounding metformin’s therapeutic effects in animal models of human disease, ranging from potential application in cardiovascular diseases and viral infections to neurological and neurodevelopmental disorders. This review delves into the origin, discovery, and early clinical applications of metformin, charting its path from an ancient herbal remedy to a drug with the potential to treat an array of human diseases.

## 2. Metformin: A Brief Overview

### 2.1. Origin, Synthesis, and First Clinical Application

The chemical origin of metformin has been traced to guanidine derivatives extracted from the medicinal plant *Galega officinalis* (also known as Spanish sainfoin, professor weed, French lilac, goat’s rue, or Italian fitch) in medieval Europe. While the guanidine compounds, galegine and synthalin, of *Galega officinalis* were reported to have a glucose-lowering effect, their acute potency impeded their use in diabetes treatment [6]. By then, experiments conducted on rabbits demonstrated the glucose-lowering effects of guanidine [7]. Subsequent studies showed that biguanides formed by the fusion of two guanidines were less toxic than the mono- and di-guanidines. Based on these findings, Irish chemists Werner and Bell pioneered the synthesis of metformin [8]. Despite its availability, it took another 36 years until French clinician Jean Stern demonstrated the effectiveness of metformin in treating adult-onset diabetes [9]. The first clinically approved application of metformin in the context of diabetes management was recorded in Europe within the same year as Sterne’s findings. Meanwhile, the U.S. FDA approved the clinical application of metformin for type 2 diabetes (T2D) in 1994 (https://www.accessdata.fda.gov/drugsatfda_docs/nda/98/020357s010_appltr_medr_chemr_EA.pdf, accessed on 3 November 2024).

### 2.2. Metformin Administration and Dosage

**Figure 1:**
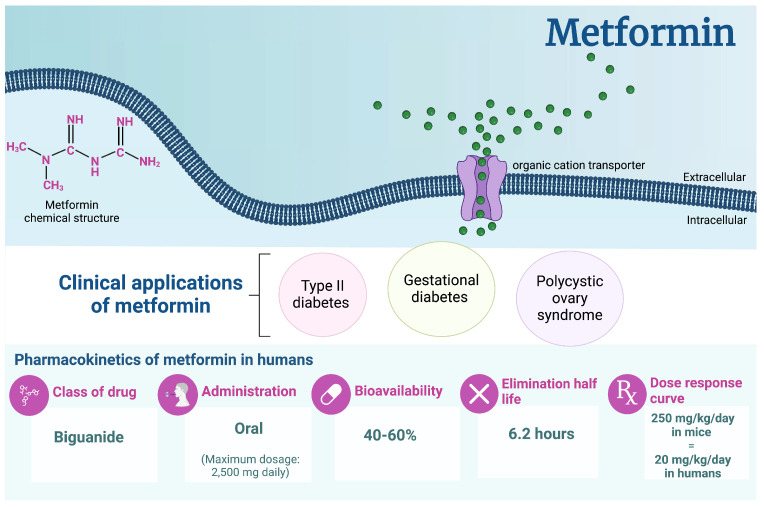
**Overview of metformin chemical structure, clinical use, and pharmacokinetics.** Chemically, metformin is a biguanide (1,1-dimethylbiguanide) used in the treatment/management of type 2 diabetes, gestational diabetes, and polycystic ovary syndrome. Metformin is administered orally in immediate- or extended-release form. Between 40–60% of metformin enters systemic circulation and is eliminated from the body in about 6.2 h. For pre-clinical studies in animal models, 250 mg/kg/day in mice corresponds to 20 mg/kg/day in humans [10,12]. Illustration created in BioRender.com.

As the preferred therapy for T2D, metformin is typically prescribed at a maximum dosage of about 2000–2500 mg/day and is orally ingested as immediate- or extended-release tablets or solutions (Figure 1). The extended-release solution of metformin hydrochloride, Riomet ER, was only approved for clinical use by the FDA five years ago. Until then, metformin in solution was only available as an immediate-release form. Riomet ER is prescribed in combination with adjustments to diet and exercise for adults and children (≥10 years) [10]. Although metformin has been widely prescribed since being approved by the FDA, some side effects have been associated with different formulations of the drug. In a retrospective chart review of 471 patients, data were gathered from selected 310 patients who received extended-release metformin and 158 patients who received immediate-release metformin. The patients who were included in this study had an average of 56 years of age with a mean body mass index of 33 kg/m^2^ (considered to be overweight), while also having diabetes. In these patients, the populations who experienced gastrointestinal (GI) side effects were close, with 11.94% and 11.39% GI side effects associated with metformin extended-release or metformin immediate-release formulations, respectively [11].

### 2.3. Pharmacokinetics and Pharmacodynamics of Metformin

Following ingestion, metformin is widely distributed in cells of our body through organic cation transporters (OCT). At intestinal pH, metformin exists as a cationic hydrophilic base and possesses physiochemical properties that hinder its passive diffusion across cell membranes [13]. While metformin absorption is believed to be primarily through plasma membrane monoamine transporters (PMAT) of enterocytes, studies have suggested a role for OCT1 and OCT3 in this process. The post-absorption plasma concentration levels of metformin range from 54 to 4133 ng/mL, but individuals with OCT1 variants have lower plasma concentrations [14].

Metformin enters hepatocytes through OCT1 and OCT3 transporters [15,16]. It is generally accepted that metformin antagonizes the 5′-AMP-activated protein kinase (AMPK) to impact cellular energy stress. It inhibits complex I of the mitochondrial electron transport chain, decreases adenosine triphosphate (ATP) production, and increases the AMP/ATP ratio, which is detected by the AMPK. Metformin-induced AMPK activation diminishes acetyl-CoA carboxylase (ACC) activity, promotes fatty acid oxidation, and hinders lipogenic enzyme expression [17]. Through AMPK activation, metformin also inhibits the mechanistic target of rapamycin (mTOR) complex 1 (mTORC1) signaling [18]. Conversely, studies also suggest that metformin exerts its effects through an AMPK-independent mechanism, inhibiting phosphatidylinositol 3-kinase/Akt/mTOR (PI3K/AKT/mTOR) pathways [19].

About 90% of absorbed metformin is eliminated within the first 24 h following oral administration, with a plasma clearance half-life of approximately 6.2 h. Meanwhile, the removal half-life of metformin in the blood is roughly 17.6 h [20]. Although metformin is not metabolized, several organs are involved in its elimination from the human body. Excretion of metformin from the liver is facilitated by organic cation antiporter multidrug and toxin extrusion (MATE) 1 [21]. Similarly, the elimination of metformin is also facilitated by the kidneys. Metformin uptake from circulation happens via OCT2 in renal epithelial cells, followed by secretion into the tubular lumen through MATE1 and MATE2 [22,23].

## 3. Current Clinical Applications of Metformin in Human Disease

The anti-hyperglycemic effects of metformin in humans have led to its application in T2D. However, metformin has also been applied in the context of other human conditions with metabolic syndromes/diseases, such as polycystic ovary syndrome (PCOS) and non-alcoholic fatty liver disease (NAFLD). Table 1 summarizes the currently approved and prescribed applications of metformin and the relevant biological pathways involved.

## 4. Current Applications of Metformin in Animal Models of Human Disease

In recent years, studies have suggested a potential role for metformin as a potential therapeutic strategy for a range of human diseases. Arguably, escalation in research into the application of metformin in cardiovascular diseases, cancer, neurological diseases, and COVID-19 may be accredited to its anti-inflammatory, anti-cancer, neuroprotective, and cardioprotective effects [36,37,38,39]. However, the biological mechanism(s) by which metformin exerts its effects remains controversial.

### 4.1. Metformin and Cardiovascular Diseases

Evidence of the potential cardioprotective effect of metformin is suggested by the inhibition of inflammation, cellular apoptosis, and autophagosome formation in the context of ischemia/reperfusion (I/R) injury associated with acute myocardial infarction (AMI) [40]. AMI results from insufficient blood flow (ischemia) to the myocardium and causes loss of viable heart tissue. Within minutes of the ischemic insult, irreversible damage to cardiomyocytes occurs *due to* the unavailability of oxygen and nutrients. Although the treatment of AMI involves the improvement of blood flow to the ischemic myocardium, this reperfusion leads to further injury by the formation of reactive oxygen species (ROS), neutrophil invasion, and calcium overload [41]. In vivo animal models of I/R injury suggest that intracellular processes, such as autophagy, are increased in AMI. While autophagy may trigger cell survival pathways, defective autophagy is detrimental [42]. Within the first minutes of AMI, autophagy has a protective effect against oxidative stress in cardiomyocytes. However, prolonged autophagy may result in cell death [43]. Thus, the modulation of the spike in AMI-associated autophagy has been considered a therapeutic approach to reperfusion injury treatment.

In one study, AMI was induced in male GFP-LC3 transgenic mice by ligation of the left coronary artery. Mice were observed 0.5, 4, and 24 h after following ligation. In another group of mice, autophagy was manipulated by the administration of bafilomycin (lysosomal inhibitor) 30 min prior to coronary ligation [44]. The results suggest that protective autophagy commences in the ischemic region within 30 min after coronary ligation and reaches its maximum at the 4 h mark. However, this protective autophagy drastically declines after 24 h. Meanwhile, bafilomycin accelerated the ischemic death of cardiomyocytes [44]. The results from another study suggest that cell death caused by excessive autophagy, or autosis, is induced by I/R in the heart of mice [45]. The researchers also found that selective suppression of autosis reduces I/R injury in cardiomyocytes [45].

Studies have shown that metformin improves cardiac function and reduces I/R injury in humans and mice [46,47]. To modulate the effect of autosis in AMI, research has further explored the cardioprotective potential of metformin. In one study, mice injected with metformin prior to ischemia induced by surgical ligation of the left coronary artery had reduced infarct size and creatine kinase (an indirect marker of muscle damage) [40]. The study found that metformin exerts its cardioprotective effects by inhibiting autophagosome formation induced by I/R injury to protect cardiomyocytes from apoptosis and inflammation. In doing so, metformin restores autophagosome processing while hindering autophagosome formation [40]. Similar results have been found experimentally by several groups [48,49]. The cardioprotective role of metformin in other biological pathways has also been supported in other studies using rats and mice. For instance, research suggests that metformin attenuates mitochondrial irregular function, mitochondrial dynamic imbalance, and apoptosis subsequent to I/R injury [50,51]. Further, the cardioprotective effect of metformin is also suggested to be mediated by AMPK phosphorylation and decreased *NOX4* gene expression [52]. Collectively, these preclinical studies support the potential use of metformin in the treatment of AMI and other cardiovascular diseases.

### 4.2. Metformin and Cancer

The anti-cancer effect of metformin is characterized by its impact on cell proliferation and survival pathways frequently perturbed in cancer, including the AMPK and PI3K/AKT pathways. Considering that the global prevalence of T2D continues to rise and that people with T2D are at greater risk for malignancies of the liver, uterus, breast, pancreas, and colon, research into the application of metformin in the context of cancer remains relevant [53,54]. Studies suggest that metformin, through the inhibition of neoplastic cell metabolism and inflammation within the tumor microenvironment, may reduce the prevalence of certain forms of cancer [55,56,57]. Under normal physiological conditions, the PI3K/AKT signaling pathway regulates proliferation, cell cycle, and apoptosis. As dysregulation of PI3K/AKT signaling is featured in tumorigenesis and proliferation of cancer cell proliferation, drugs targeting PI3K/AKT signalling, its downstream, or its upstream components have been attractive therapeutic avenues [58]. In this regard, metformin inhibits tumor progression and promotes cell cycle arrest by disrupting the PI3K/AKT pathway in endometrial, colorectal, and prostate cancers [58].

The correlation between metformin administration and reduced risk of carcinogenesis has long been observed [59]. Now, researchers are actively trying to counteract carcinogenesis with metformin, and initial success can be attributed to several causes. Firstly, the inhibition of the respiratory complex leads to an increased production of ROS, which in turn can trigger DNA damage in the affected cells. In this way, metformin directly contributes to an increased apoptosis rate [60]. Inhibition of complex I also strongly interferes with the energy balance of the cell. Cancer cells that rely solely on ATP production for their metabolism cannot compensate for the sudden loss of ATP and also undergo apoptosis [60].

*Due to* the resulting shift in the AMP/ATP ratio, the central signal transduction molecule AMPK is phosphorylated and thus activated by the known tumour suppressor LKB1, which in turn results in a range of downstream effects. The antiproliferative effects of LKB1 are mainly based on the inhibition of global protein and lipid synthesis, arrest of cell growth and cell cycle progression, and reduced angiogenesis. AMPK plays a major role in the energy regulation of the cell and brings all anabolic processes of the cell, such as protein or lipid synthesis, to a standstill and consequently stops cell growth [61].

A crucial pathway is PI3K/AKT/mTOR signal transduction, which is often deregulated in human malignancies [62]. The ultimate activation of mTOR in a healthy cell leads to increased activation of various key proteins of the translational machinery, such as eukaryotic translation initiation factor 4 gamma 1 (eIF4G) and S6 kinases [60,62]. In addition, mTOR plays a major role in the negative feedback loop of insulin signalling. If this signal is suppressed, the sensitivity of the cell to insulin stimulation increases, which again counterbalances the energy status. Metformin also directly suppresses mTOR signalling via the inactivation of Rag GTPases and the upregulation of regulated in development and DNA damage responses 1 (REDD1) [60,61].

The mTOR kinase is only one of the many downstream molecules of the key enzyme AMPK. Extremely relevant in the context of brain tumours is its influence on the tumour suppressor p53, which is frequently mutated, especially in glioblastomas [60]. By activating AMPK, metformin also ensures increased autophagy and apoptosis rates via this mediator [60].

Metformin affects a number of signalling pathways with important roles in the further differentiation of cancer stem cells. By suppressing the sonic hedgehog (Shh) pathway at both transcriptional and translational levels, processes such as epithelial-to-mesenchymal transition (EMT) or neovascularisation are significantly reduced, as shown in in vitro experiments with pancreatic carcinoma cells [63]. In non-small cell-lung cancer cell lines, an active reversal of the EMT process by metformin could even be proven, as demonstrated by EMT marker (E-cadherin, vimentin) levels. A reduced ability of the cells towards migration was also demonstrated, which can be attributed to the presumed connection between TGFβ and migration or metastasis of the cancer cells. This effect is probably achieved primarily through the interaction of metformin with TGFβ1, preventing heterodimerisation with TGFβR2 and, thus, the successful metastasis of the cancer cells [64].

### 4.3. Metformin, COVID-19, and Other Viruses

Studies suggest that metformin disrupts various stages of the viral life cycle and impacts the host cell’s lipid metabolism to exert its antiviral effect. The antiviral mechanism is proposed as an activation of AMPK in the early stages of infection by some viruses, resulting in a reduction of intracellular lipids [65]. In recent years, the effect of metformin in viral contexts, including DNA viruses (human papillomavirus, herpes simplex virus, Kaposi’s sarcoma-associated herpesvirus, and hepatitis B virus) and RNA viruses (Rotavirus, Dengue, Zika, yellow fever, severe acute respiratory syndrome coronavirus-2, hepatitis C, influenza A, and human immunodeficiency viruses) has been explored. However, the precise mechanism(s) by which metformin applies its antiviral effect is yet to be defined. For instance, metformin has been suggested to inhibit the replication of DNA viruses by inhibiting the repression of viral transcription genes [65]. The effect of metformin on viral infection has been covered in different articles [66,67,68].

## 5. Dysregulated Brain Metabolism Is an Avenue for Application of Metformin in Neurological Disorders

The human brain relies almost exclusively on glucose as its source of energy under normal circumstances [69]. The adult brain is exceptional as it consumes approximately 20% of the glucose available to the body while only accounting for 2% of the body weight [69,70]. Glucose is used to produce ATP, a high-energy molecule, by undergoing glycolysis, the tricarboxylic acid (TCA) cycle, and oxidative phosphorylation. ATP is used to manage action potentials, synaptic transmission, and other cellular functions [71]. Glucose needs to be highly regulated to ensure proper brain health, as dysregulation of glucose metabolism has been associated with disease processes. Glucose enters the brain via glucose transporters (GLUTs), specifically GLUT1 and GLUT3 [69]. GLUT1 ensures adequate glucose transport to glial cells of the brain, whereas GLUT3 is responsible for providing glucose to neurons since it has a faster rate of transport [69]. These glucose transporters act independently of insulin, ensuring a continuous supply of glucose to the brain [72]. GLUT1 and GLUT3 have even been demonstrated to be upregulated in the brain when glucose is low, allowing the brain to use the limited glucose [72]. There are times when glucose may not be as readily available to the brain to be used as an energy source. This situation commonly arises during times of fasting, and ketones help fulfill the energy requirements of the brain while glucose is not accessible. Free fatty acids can be processed into ketone bodies, which can cross the blood–brain barrier (BBB) and be used as a replacement energy source in the brain during an extremely fasting state [70]. They can cross the BBB via monocarboxylate transporters (MCTs), which become upregulated during times of fasting [73]. Ketone bodies can be broken down in the same process as glucose, but enter at the stage of Acetyl-CoA incorporation [73].

As mentioned earlier, the brain consumes ~20% of the glucose available to the body [69]. Since glucose has such a critical function in the maintenance of brain function, we can appreciate how damaging a drop in glucose metabolism would be for the human brain. Even experiencing hypoglycemia for a short period of time can have detrimental consequences for our brain, such as in the case of a stroke. However, stroke is not the only time that there is a mismanagement of glucose. Many neurodegenerative diseases have also been noted to have problems surrounding the availability of glucose for the brain. For example, Alzheimer’s disease (AD) is a condition that is shown to be associated with a decreased ability to process and metabolize glucose, leading to increased glucose concentrations in the brain [74]. GLUT3 levels in AD are much lower compared to healthy controls, preventing glucose uptake in the brain and, therefore, decreasing the ability of the brain to perform optimally [74]. Interestingly, T2D mellitus (T2DM) increases the risk of developing AD, proposing a further link between glucose metabolism regulation and brain impairment [75]. T2DM has been shown to result in changes in brain structure and cognitive impairment [76]. Specific brain areas such as the hippocampus, basal ganglia, and the orbitofrontal cortex proved to have significantly smaller sizes in those patients with T2DM compared to controls, though total brain volume and grey matter areas were also significantly decreased [76]. In terms of brain function, memory, speed processing, and executive function were all drastically reduced in T2DM individuals compared to controls [77]. In comparison to AD and T2DM, where decreased metabolism is the source of the impairment, Parkinson’s disease (PD) results from hypermetabolism issues in the external pallidum and hypometabolism throughout the cortex [78]. PD is characterised by movement abnormalities, including resting tremor, rigidity, postural imbalances, and bradykinesia [79]. The mitochondria in PD are noted to be altered, specifically in the electron transport chain (ETC) [79]. According to Mizuno et al., complex I of the ETC contained more than one impaired subunit in PD individuals and also had reduced function compared to controls [80]. Therefore, this once again demonstrates that mitochondrial function involved in glucose processing can have devastating impacts on the brain and the individual overall.

**Figure 2:**
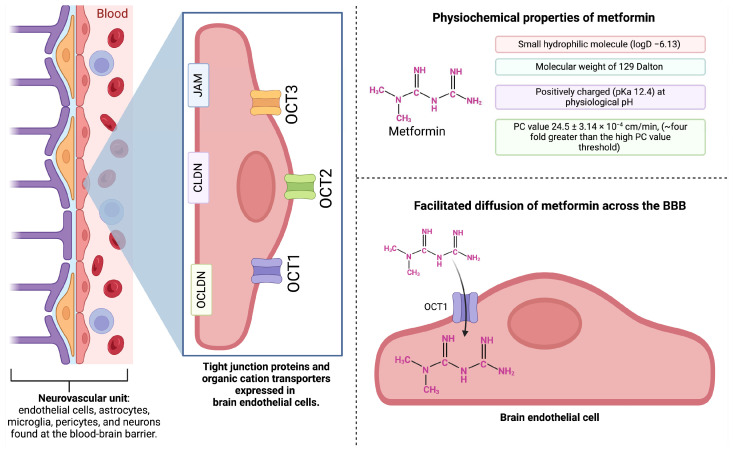
**A proposed mechanism for the entry of metformin into the brain via the blood–brain barrier (BBB).** The BBB is comprised of neurovascular units composed of brain endothelial cells, supporting cells (astrocytes, microglia, pericytes, and neurons), and tight junctional proteins. Endothelial cells of the neurovascular unit possess OCT1 transporters, which metformin can use to pass through the BBB. The physicochemical properties of metformin (small, hydrophilic, and positively charged molecule) suggest that metformin may use OCT for transporting across the BBB [83,84]. CLDN: claudins, OCLDN: occlusins, JAM: junction adhesion molecules, OCT: organic cation transporters, PC: permeability coefficient. Illustration created with BioRender.com.

Given that metformin is suggested to cross the BBB (unassisted) and that neurometabolic impairments are featured in some neurological conditions, metformin proves to be an appealing candidate for the treatment and/or management of neurological conditions. While the three OCT subtypes (OCT1, OCT2, and OCT3) have been reportedly present in the mammalian brain, studies suggest that OCT1 and OCT2 specifically function to permit the entry of metformin into the brain [81]. However, in vivo and in vitro experiments detailing the lack of expression of OCT in mouse, rat, or human brain microvessels challenge the presence of OCTs in all mammals [82]. The physiochemical properties of metformin (small hydrophilic molecule, pKa 12.4 and positively charged at physiological pH, and molecular weight of 129 Dalton) suggest that its transport is driven by rapid passive diffusion (Figure 2) [83]. BBB permeability studies in an in vitro co-culture model of BBB suggest that OCT1 is the primary transporter used to transport metformin across the BBB [83].

## 6. Metformin and Neurological Disorders

The metabolic impairments associated with various neurological conditions have made metformin an attractive potential therapy for the treatment of these diseases. Over the years, research into the application of metformin in models of neurological diseases has progressed, and the results of recent findings are summarized in Table 2.

### 6.1. Metformin as a Potential Therapeutic Avenue for Neurodevelopmental Diseases

Although there are limited reports on the effects of metformin on neurodevelopmental disorders, there have been increasing attempts in recent years to study the neuroprotective properties of this anti-diabetic drug.

The aetiology of Rett syndrome (RTT), a rare neurodevelopmental disorder (discussed later), is mainly based on de novo mutations of the methyl CpG binding protein 2 (*MECP2*) gene, which encodes for a transcription factor mediating gene regulation [92]. In one study, researchers observed aberrant mitochondrial activity and oxidative stress in a female mouse model of RTT. Zuliani et al. conducted a study in 2020 in which they tested the effectiveness of metformin in the context of deficient mitochondrial activity in the RTT mouse model [93]. The results suggested that aberrant mitochondrial biogenesis was fully restored, as measured by ATP levels in the brain and periphery. Furthermore, the levels of various proteins involved in mitochondrial biogenesis and remodelling, which are classically only expressed to a significantly reduced extent in RTT, were largely raised to control levels. These include PGC-1alpha, which is activated as a direct target of metformin, as well as OPA1 and MFN2, which play a key role in the dynamic remodelling process. Metformin stimulates the antioxidant PGC-1α/Nrf2/HO-1 signalling pathway and significantly improved oxidative stress levels in female mice [93]. An in vivo study from our group demonstrated that metformin significantly *increases MECP2* transcripts in an isoform-specific manner. *BDNF* (brain-derived neurotrophic factor), which especially influences hippocampal neurogenesis as a growth factor, is also disturbed in its homogenesis in RTT and, similar to *MECP2*, can be increased by metformin [94]. Our group further reported that metformin partially relieves the inhibitory effect of the MeCP2 isoform, MeCP2E1, on the *Mecp2* promoter in Daoy medulloblastoma brain cells [95]. Together, these studies suggest a role for metformin in ameliorating the effect of de novo mutations of the *MECP2* gene.

In addition, the connection of the mTOR and downstream signaling pathways to RTT have been studied in the context of patient brain tissues [96,97]. This includes fragile X syndrome, a monogenetic disorder caused by the loss of the *Fmr1* gene of the X chromosome that leads to social behaviour problems, learning difficulties, and developmental delays, collectively known as ASD [98]. The loss-of-function mutation leads to hyperactivation of the mTORC1 and ERK signalling pathways, both of which can be inhibited by metformin [18]. Gantois et al. found that metformin indeed had a positive effect on the phenotype not only at the molecular level (restoration of several dysregulated proteins such as p-ERK, p-eIF4E, and MMP-9) but also at the functional level (improved social deficits significantly) [91]. Clinical trials on the efficacy of metformin in relation to fragile X syndrome have recently started. Initial case studies generally show a positive trend, but they have been terminated [99,100]. Additional clinical trials are in progress: https://clinicaltrials.gov/ct2/show/study/NCT03479476, accessed 3 November 2024; and https://www.clinicaltrials.gov/ct2/show/NCT03862950, accessed 3 November 2024.

### 6.2. In Vivo and in Vitro Studies and Clinical Trials of Metformin Application in Brain-Related Complications

#### 6.2.1. Neurorepair and Neurogenesis

Metformin achieves its neuroprotective effects, besides others, through its ability to stimulate endogenous mechanisms for neurorepair. By overstimulating neural stem cells, an attempt is made to stimulate their proliferation and differentiation and thus counteract the pathological demise of neurons and glial cells. As early as 2012, Wang et al. postulated the connection between metformin and increased neurogenesis in the adult forebrain. In in vivo experiments, the team showed that the number of Ki67+ neural stem cells in the subventricular zone increased significantly after metformin administration. Hippocampal neurogenesis is also increased after metformin therapy, as demonstrated by immunostaining against various newborn neuronal markers; however, the neural stem cell pool remains unchanged [101]. This association has many applications: metformin treatment led to increased executive functions in clinical trials of neurodegenerative diseases such as AD, although there was no improvement in the corresponding biomarkers [102,103]. A phase three trial is currently underway to shed further light on these effects (https://clinicaltrials.gov/ct2/show/NCT04098666?term=metformin&cond=Alzheimer+Disease&draw=2&rank=1, accessed 3 November 2024). Treatment with metformin-induced endogenous neurogenesis is also tempting in related diseases such as PD, which is caused by direct destruction of dopaminergic neurons, although clinical data are not yet available [104,105].

Another cause of premature neurodegeneration is radiation therapy for brain tumours. Especially in children, radiation-induced damage to the hippocampus and white matter leads to reduced neurogenesis, whereby the neural stem cell pool in areas such as the dental gyrus does not recover on its own [106]. Ayoub et al. modelled this situation in a mouse model and achieved a complete recovery of the stem cell pool after metformin treatment, starting 1 day after radiation [106]. Similar results were observed in vivo in a model for hypoxia/ischemia induced injury. Remarkably, metformin increases not only the relative proportion of neuronal precursor cells but also the absolute number of cells [107].

Functionally, these effects of metformin can be determined by using various behavioural tests in mice. The most suitable tests are those involving memory. Memory formation is characterised by a high degree of neuroplasticity in the hippocampus, which is why changes affecting the neurogenesis rate are directly reflected in the behaviour. Ayoub et al. conducted tests in a mouse model of radiation-induced damage, whereby all deficits could be compensated for in a sex-specific manner by metformin treatment (post-radiation) [106]. As early as 2012, Wang et al. demonstrated increased spatial memory after metformin treatment, correlating with increased neurogenesis in the hippocampus [101,103]. Derkach et al. studied olfactory memory in rodents, achieving full recovery of olfactory memory along with increased neuroblast proliferation by pre-emptive treatment with metformin prior to radiation-induced injury [108].

#### 6.2.2. Inflammation

In addition to increased neurogenesis, metformin also suppresses the development of inflammation in the brain, which typically accompanies the degeneration of neurons. The inhibition of nuclear factor kappa-light-chain-enhancer of activated B cells (NF-κB) suppresses the release of chemokines, which in turn prevents the activation of microglia and astrocytes and reduces the release of proinflammatory cytokines (TNFα, IL-6, IL1β) and ROS, respectively [109]. Hassan et al. demonstrated the beneficial effects of metformin in sepsis-associated encephalopathy in rats. Metformin treatment significantly reduced the measurable levels of proinflammatory cytokines such as high mobility group box 1 protein (HMGB1) and improved sepsis-impaired BBB integrity by enhancing the expression of significant tight junction proteins [110]. Taken together, these neuroprotective effects of metformin provide broad protection against a range of diseases.

### 6.3. Proposed Mechanisms of Metformin Action in the Brain

**Figure 3:**
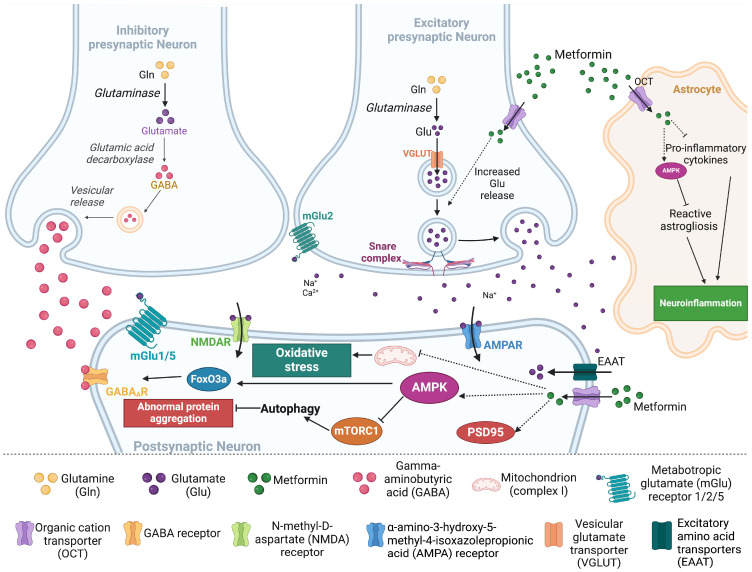
**Proposed mechanism of metformin action in neurons and astrocytes of the mammalian brain.** Metformin entry into excitatory pre- and post-synaptic neurons, as well as astrocytes, may be mediated by OCT. In excitatory pre-synaptic neurons, metformin may increase glutamate (Glu) release while mitigating reactive astrogliosis and the release of pro-inflammatory cytokines from astrocytes. In post-synaptic neurons, metformin may alter the expression of synaptic markers (such as PSD95), inhibit complex I of the mitochondrial electron transport chain, inhibit mTORC1, and mediate AMPK-dependent activation of FoxO3a. Metformin-mediated activation of FoxO3a may promote the insertion of GABA receptors at the post-synaptic membrane as an indirect effect of the drug, involving inhibitory presynaptic neurons. Adapted from Bak et al., 2006 and Li et al., 2022 with additional information extracted from Barini et al., 2016, Oner et al., 2024, Yoval-Sanchez et al., 2022, Fan et al., 2019 [111,118,119,120,121,122,123]. Abbreviations: AMPK: 5′ adenosine monophosphate (AMP)-activated protein kinase; FoxO3a: forkhead box O3a; GAD: glutamate decarboxylase; mTORC1: mechanistic target of rapamycin complex; PSD95: postsynaptic density protein 95; SNAP25: synaptosomal associated protein 25. Illustration created with BioRender.com.

An accumulating body of evidence suggests that metformin may regulate synaptic transmission and plasticity (Figure 3) [111]. After treating acute hippocampal slices from male C57BJ/6 mice with varying concentrations of metformin, researchers found that metformin increased the miniature excitatory postsynaptic currents (mEPSC) frequency. However, neither the frequency nor the level of miniature inhibitory postsynaptic currents (mIPC) were changed following metformin treatment [112]. This contrasting effect in mEPSC and mIPCs led researchers to conclude that glutamatergic transmission is enhanced in hippocampal CA1 pyramidal neurons, while GABAergic transmission is not altered [112]. Further, intrathecal treatment with metformin repressed the frequency of spontaneous excitatory postsynaptic currents (sEPSC) in neurons of the spinal dorsal horn of male Sprague–Dawley rats [113]. Moreover, studies in a fragile X syndrome mouse model suggest that metformin ameliorated the aberrant synaptic release and the Munc18-1 accumulation in *Fmr1*-KO neurons and inhibited the translation of synaptic proteins in presynapses [114]. In AD mice, chronic metformin treatment restored spatial memory, long-term potentiation expression, dendritic spine density, and surface GluA1 trafficking [115]. Together, these studies indicate that metformin exerts varying effects on presynaptic neurons.

It is generally accepted that glial cells, such as astrocytes and microglia, play key roles in neuroinflammatory cascades of neurodegenerative diseases; the role of metformin in astrocytes and microglia in the context of neuroinflammation has thus been investigated. Studies in primary cultures of rat astrocytes revealed that metformin reduced the release of cytokines, among other effects [116]. The results from other investigations suggest that metformin attenuates epilepsy-induced microglial activation and inhibits astrogliosis in a rat model of temporal lobe epilepsy [87]. Further, the metformin-modulated inflammatory response in hypothalamic astrocytes of an immunocompromised mouse model induces expression of transcription factors known to regulate the inflammatory response [117]. Despite the many studies on the effect of metformin in the brain, a validated mechanism of metformin’s neurological action is yet to be fully established.

## 7. Metformin and Epigenetics

### 7.1. A Brief Overview of Epigenetics

Direct genetic changes in our DNA sequence are responsible for many disorders and diseases. However, alterations in the expression of our genes are another way in which diseases can present, including neurodevelopmental conditions. Many brain-related conditions are associated with changes in epigenetics, including autism spectrum disorder (ASD), fetal alcohol spectrum disorders (FASD), Angelman syndrome, Rett syndrome, Prader–Willi syndrome, and others [124,125,126]. Common methods for controlling gene expression include methylation of the DNA, histone modifications, and the use of microRNA (miRNA). Another difference between DNA modifications and epigenetic changes is the fact that the latter can be reversible. Problems arise when epigenetic control is altered [127]. MeCP2 is one example of this troublesome scenario, which will be explained in detail below. Other neurodevelopmental conditions have been linked to epigenetics, including those listed above, though some neurodegenerative disorders are also known to have epigenetic causes, such as HD, PD, and AD [128].

#### 7.1.1. Rett Syndrome and Epigenetics

Rett syndrome is one of the most notable conditions in young children in which the control of gene expression is critically altered *due to* a mutation in the *MECP2* gene. The protein encoded by this gene, MeCP2, binds to methylated CpG sites in the DNA and contributes to repressing or activating gene expression and resisting the effects of nucleases in these areas [129]. Transcription from methylated regions of DNA has been shown to be strongly repressed in the presence of MeCP2, whereas unmethylated regions were not repressed [130]. Therefore, MeCP2 may have significant impacts on the regulation of methylated regions of DNA, and its absence or compromised function can affect human health conditions [131]. RTT, in the majority of cases, is attributed to alterations in this gene, resulting in numerous harmful and life-threatening symptoms.

#### 7.1.2. Autism Spectrum Disorders (ASD) and Epigenetics

ASD cover a wide range of conditions that present with sparse language abilities, greatly decreased social capabilities, and repetitive behaviours [132]. ASD are also connected to the altered expression of MeCP2. However, other genes have been epigenetically linked to ASD, including the oxytocin receptor gene (*OXTR*) [133]. Oxytocin is a hormone in the body that is responsible for many social factors in everyday life, such as the ability to express emotion, bonding with others, and social memory, recognition, and behaviour [133]. Oxytocin is of interest in ASD since one of the main deficiencies in the condition is the lack of proper social skills. Modahl et al. discovered significantly lower levels of oxytocin in plasma in children with autism compared to those who were developing normally, further establishing the connection between oxytocin and social interaction abilities [134]. *OXTR* has been shown to be impacted by both genetic and epigenetic modifications, both of which are linked with autism [135]. In that study, two children diagnosed with autism were born from the same mother. Although one child had a deletion mutation in *OXTR*, the other child had *OXTR* silenced via DNA methylation [135]. Thus, there may be multiple epigenetic mechanisms that contribute to a single disorder.

### 7.2. Effects of Metformin on the Epigenetic Landscape of the Brain

Metformin has been shown to exert profound effects on the epigenetic landscape of various tissues, including the brain. In mice, metformin treatment induces significant changes in histone modifications, including histone composition and post-translational modifications in brain tissue.

#### 7.2.1. Metformin and Histone Modifications

Metformin treatment leads to notable alterations in histone acetylation patterns in the murine brain. It significantly upregulates the expression of *BDNF* by increasing histone acetylation at its promoter [136]. This increase in histone acetylation is thought to promote an open chromatin state, potentially enhancing the expression of neuroprotective genes. Such impact on histone acetylation is partly mediated through its effects on histone deacetylases (HDAC). Metformin activates AMPK, which influences histone acetylation by regulating histone acetyltransferases (KATs) [137]. This modulation contributes to the overall increase in histone acetylation and may play a role in metformin’s neuroprotective effects. Metformin has been shown to influence various histone modifications, which play an important role in the epigenetic regulation of gene expression.

Further, metformin influences histone methylation by decreasing the levels of repressive histone marks, such as H3K9me2 and H3K27me3, while increasing active marks, such as H3K4me3 [138]. In one study, metformin suppressed the increased level of histone H3K36me2 associated with the development of pre-diabetes [139]. Metformin affects the activity of histone-modifying enzymes. For instance, it inhibits the activity of certain HDAC proteins, such as HDAC2 and HDAC3, in various tissues [138]. Metformin also stimulates the activity and expression of sirtuins, especially SIRT1 and SIRT6, which are NAD^+^-dependent deacetylases that regulate cellular metabolism and energy homeostasis [137,140].

Recent studies have revealed that metformin can alter H2A.Z dynamics and regulate gene expression, particularly in the context of prostate cancer cells. This finding shed light on potential mechanisms through which metformin may exert its anti-cancer effects beyond its primary role in diabetes management. In androgen-dependent androgen receptor (AR)-positive LNCaP cells, metformin treatment increases H2A.Z occupancy on the AR gene and AR-regulated genes [141]. Interestingly, this increase in H2A.Z incorporation is predominantly attributed to the H2A.Z.1 isoform. The specificity of this effect was confirmed through siRNA-mediated knockdown experiments, which identified H2A.Z.1 as the primary isoform responsible for the observed change in gene regulation following metformin treatment. The modulation of H2A.Z dynamics by metformin appears to be part of broader epigenetics in cancer cells [141]. Research suggests that metformin effects on early stages of prostate cancer may involve histone methyltransferase EZH2 and H2A.Z, potentially altering different molecular pathways. This multi-faceted epigenetic impact underscores the complexity of metformin action at the chromatin level. By changing the levels and gene-binding dynamics of histone variant H2A.Z, metformin may target prostate cancer cells [141]. This mechanism of action provides new insights into how metformin might exert its potential anti-cancer effects. The drug’s ability to modulate H2A.Z incorporation and distribution, particularly of the H2A.Z.1 isoform, suggests a pathway through which metformin could influence gene expression and cellular behaviour in the context of cancer [141].

#### 7.2.2. Metformin and Epigenetic Regulators

Research indicates that metformin treatment affects epigenetic regulators in murine brain tissue. For instance, it induces MeCP2 in the hippocampus of male mice [142]. This alteration in epigenetic regulators may contribute to its potential cognitive-enhancing effects. It is important to note that metformin impacts histone composition uniformly in the brain. Studies from our team have revealed varying effects in different brain regions, with the hippocampus showing particularly pronounced changes in a sex-dependent manner [142]. This regional specificity may underlie varied effects of metformin on certain cognitive and behavioural processes. In placental tissue, metformin increases histone acetylation (H3K27ac). This was shown in human placental explants that originated from male offspring [143].

The metformin-induced changes in histone post-translational modifications in the mouse brain have been linked to its neuroprotective effects and potential improvements in cognitive function. These epigenetic alterations are associated with upregulation of genes involved in synaptic plasticity and neurogenesis [136].

**Figure 4:**
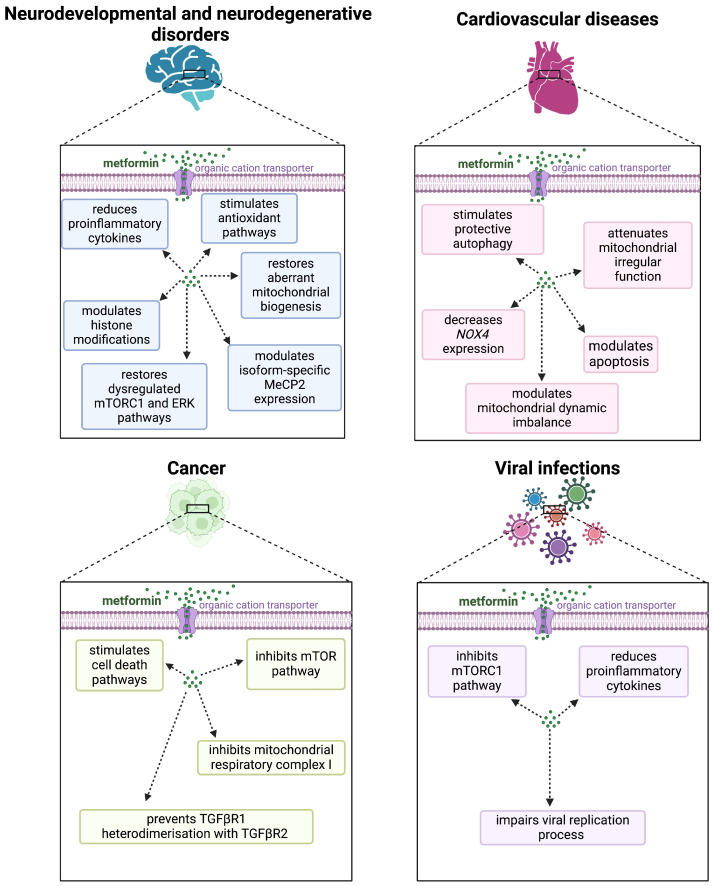
**Summary of the potential effect of metformin in different human diseases.** The pleiotropic effects of metformin are observed in the context of different diseases, including neurological and cardiovascular diseases, as well as in cancer and viral infection. Metformin may be a promising therapeutic agent in various diseases. Information obtained from: [18,40,46,47,48,49,50,51,52,59,61,65,91,93,94,95,109]. Illustration created in BioRender.com.

Overall, metformin exerts multifaceted effects on the epigenetic landscape of the murine brain, particularly influencing histone acetylation and epigenetic regulators. These changes have significant implications for gene expression patterns related to neuroprotection and cognitive function, highlighting the potential of metformin as a neuromodulatory agent beyond its established role in diabetes management (Figure 4) [144].

## 8. Limitations of Repurposing Metformin as an Alternative Therapy for Human Diseases

Typically, drug–drug interactions are clinically relevant in assessing their effectiveness. For instance, proton-pump inhibitors and other anti-diabetic drugs, such as rosiglitazone and epaglinide, inhibit OCT and their ability to uptake metformin [145,146]. The cautionary tale of using metformin, however, is specifically related to its pharmacogenomics. Genetic polymorphisms in the genes of transporters primarily involved in metformin uptake and elimination may directly impact its pharmacokinetics. Loss-of-function variants of *OCT1* are shown to lower metformin response in mouse models and in certain patients [147]. Similarly, genetic variants of *OCT3* may also impact metformin uptake, while variants of *MATE2* affect metformin elimination [148]. Thus, despite its potential therapeutic effect in a plethora of human diseases, the effectiveness of metformin may depend on the presence or absence of genetic polymorphisms in the genes of the *OCT* or *MATE* transporters in a given individual.

## 9. Preclinical to Clinical Translation of Metformin in Non-Diabetes Contexts

Compared to other drugs that treat T2D (sulfonylureas, glitazones, glinides, gliptins, and gliflozins), metformin is the most widely prescribed drug with generally few adverse side effects. Additionally, the affordability of metformin relative to other anti-diabetic medications on the market makes it a better candidate for drug repurposing. It is also important to note that location, pharmacy, dosage, quantity, insurance, or specific drug formulation may affect the cost of the drug. Meanwhile, the plethora of scientific investigations into diverse applications of metformin in non-diabetic contexts with promising results further supports the potential repurposing of the drug. However, the exact mechanisms of metformin’s therapeutic effects in diseases such as cancer, viral infections, cardiovascular disease, and neurological disease require further elucidation. As mentioned earlier, metformin relies on the kidneys for elimination from the body. Inefficient clearing of metformin from the body through the kidneys increases the likelihood of lactic acidosis, the acidification of the blood *due to* a buildup of lactate in the bloodstream [149]. Thus, special care must be taken if metformin is considered as an alternative therapeutic strategy or in combination with other drugs in diseases involving renal impairment. The following table outlines some of the ongoing and completed clinical trials involving metformin in different disease contexts (Table 3).

## 10. Closing Remarks

Development of metformin stands as a remarkable example of how ancient herbal knowledge and modern pharmacological research can converge to create life-changing medical therapies. From its roots in the medieval use of *Galega officinalis* to the systematic study of guanidine derivatives, metformin history reflects the persistence and innovation of scientists who sought to turn a potentially toxic substance into a therapeutic marvel. Through decades of research and trials, what was once a dangerous compound became a safe and effective treatment, offering millions of patients with type 2 diabetes a reliable means of controlling their blood glucose levels.

Today, metformin is one of the first lines of treatment for diabetes, not only because of its efficacy in lowering blood sugar but also *due to* its relatively low cost, favorable safety profile, and additional benefits, such as cardiovascular protection. The story of metformin development underscores the importance of interdisciplinary collaboration in medicine, combining the fields of botany, chemistry, and clinical research to address one of the most pressing public health concerns of our time. As researchers continue to explore new applications of metformin, its legacy as a cornerstone of type 2 diabetes management is set to endure, highlighting metformin’s profound and ongoing impact on global health.

## Figures and Tables

**Table 1:** Currently approved and prescribed applications of metformin with relevant biological pathways.

Human Disease	Effect of Metformin	Biological Pathway Involved
Type II diabetes (T2D)	Reduced hepatic gluconeogenesis [24]	Inhibition of mitochondrial respiratory chain complex I [25]
	Reduced intestinal glucose absorption [26]	AMPK phosphorylation and activation [27]
	Increased glucose uptake and utilization [28]	
Polycystic ovary syndrome (PCOS)	Increased menstrual cyclicity and induces ovulation [29]	Reduction of androgen production by inhibition of mitochondrial complex I [30] PI3K/AKT and upregulation of SHBG and HNF-4α [31]
	Improves the underlying insulin resistance and promotes weight loss [32]	
Non-alcoholic fatty liver diseases (NAFLD)	Reduced body weight and markers of insulin resistance [33]	ApoA5 [34] and inhibition of SCD1 expression [35]
	Reduced accumulated hepatocyte and plasma triglycerides [34]	

**Table 2:** Applications of metformin in animal models of neurological diseases.

Neurological Condition	Model	Metformin Administration	Results	References
Alzheimer’s disease (AD)	3× Tg-AD mice (male, female)	200 mg/kg by intraperitoneal injection (14 days) in drinking water (4 mg/mL) starting at the age of 7–8 months for a period of 6 weeks.	Metformin restored neurogenesis and spatial memory deficits of AD in mice.	[85]
Parkinson’s disease (PD)	N-methyl-4-phenyl-1,2,3,6-tetrahydropyridine(MPTP)/p-induced PD mouse model (male, 16–18 weeks)	100 mg/kg (2 weeks) dissolved in drinking water.	Metformin delayed astrocyte senescence and prevented neurodegeneration.	[86]
Temporal lobe epilepsy (TLE)	Wistar rats (male, 180–200 g)	200 mg/kg by oral gavage.	Metformin improved TLE associated cognitive impairment by inhibiting neuroinflammation and neurodegeneration. Metformin inhibited TLE-associated microglial and astroglial activation.	[87]
Huntington’s disease (HD)	*Caenorhabditis elegans*	150 or 2000 μM in *E. coli* strain OP50 food source.	Metformin reduced polyglutamine-induced toxicity.	[88]
Amyotrophic lateral sclerosis (ALS) and frontotemporal dementia (FTD)	*C9orf72* ALS/FTD BAC mice	5 mg/mL metformin in the drinking water (for 3 months starting at 2 months of age, and for 4 months starting at 6 months of age).	Metformin improves ALS/FTD phenotypes.	[89]
Multiple sclerosis (MS)	C57/BL6 mice	250 mg/kg or 500 mg/kg in drinking water (female, for 2.5 weeks starting at 8 weeks of age).	Metformin increases oligodendrocyte AMPK activation and oligodendrocyte differentiation.	[90]
Fragile X syndrome (FXR)	*Fmr1*^−/y^ mice	200 mg/kg bodyweight/day intraperitoneal injection (males, 10 days).	Metformin rescues phenotypes and normalizes ERK signaling, eIF4E phosphorylation and MMP-9 expression.	[91]

**Table 3:** Different applications of metformin in clinical trials involving different human diseases. Information retrieved from https://clinicaltrials.gov/ (accessed 3 November 2024).

	Clinical trails *	Metformin Dose	Study Phase	Objective/Results	Status
**Cancer**	Metformin Hydrochloride and Doxycycline in Treating Patients With Localized Breast or Uterine Cancer (NCT02874430)	Unspecified	Phase 2	To investigate metformin and doxycycline combined action in cells expressing Caveolin-1 in cancer.	Active
	Metformin Hydrochloride in Preventing Breast Cancer in Patients With Atypical Hyperplasia or In Situ Breast Cancer (NCT01905046)	850 mg	Phase 3	Studying cytological atypia in unilateral or bilateral random periareolar needle aspiration.	Active
	Metformin and Chemotherapy in Treating Patients With Stage III-IV Ovarian, Fallopian Tube, or Primary Peritoneal Cancer (NCT02122185)		Phase 2	Investigating the effect of metformin in standard adjuvant or neoadjuvant chemotherapy in non-diabetic subjects with stage III-IV fallopian tube, ovarian, primary peritoneal, or carcinoma.	Active
	Chemotherapy and Radiation Therapy With or Without Metformin Hydrochloride in Treating Patients With Stage III Non-small Cell Lung Cancer(NCT02186847)	500 mg–1000 mg	Phase 2	Determining whether metformin hydrochloride with chemoradiotherapy improves survival of patients with non-small cell-lung cancer.	Active
**Alzheimer’s Disease**	Metformin in Amnestic Mild Cognitive Impairment (MCI) (NCT00620191)	1000 mg	Phase 2	Studying cognitive function improvement in Alzheimer’s disease patients using relevant biomarkers of Alzheimer’s disease [150].	Completed
	Effect of Insulin Sensitizer Metformin on AD Biomarkers (NCT01965756)	500 mg–2000 mg/day	Phase 2	Found evidence of improved executive functioning following metformin treatment as well as trends of improvement in memory, learning, and attention [102].	Completed
**Parkinson’s Disease**	Clinical Study to Evaluate the Possible Efficacy of Metformin in Patients With Parkinson’s Disease (NCT05781711)	500 mg	Phase 2	To be determined	Recruiting
**Huntington’s Disease**	TEsting METformin Against Cognitive Decline in HD (NCT04826692)	425 mg–850 mg	Phase 3	To be determined	Unknown
**Amyotrophic Lateral Sclerosis**	Safety and Therapeutic Potential of the FDA-approved Drug Metformin for C9orf72 ALS/FTD (NCT04220021)	500 mg–2000 mg	Phase 2	Evaluating tolerability and safety of metformin in participants with C9orf72 ALS.	Active, not recruiting
**Multiple Sclerosis**	Drug Repurposing Using Metformin for Improving the Therapeutic Outcome in Multiple Sclerosis Patients (NCT05298670)	1000 mg twice daily	Phase 2	To be determined	Recruiting
	Metformin Add-on Clinical Study in Multiple Sclerosis to Evaluate Brain Remyelination And Neurodegeneration (NCT05893225)	850 mg twice or thrice a day	Phase 2	To be determined	Recruiting
	Metformin Treatment in Progressive Multiple Sclerosis (NCT05349474)	500 mg–2000 mg/day	Early Phase 1	To be determined	Recruiting
**Fragile X Syndrome**	Metformin in Children With Fragile X Syndrome (NCT05120505)	50 mg–1 or 2 g per day	Phase 4	To be determined	Recruiting
	A Trial of Metformin in Individuals With Fragile X Syndrome (Met) (NCT03862950)	250 mg–2000 mg	Phase 2	To be determined	Recruiting
	A Trial of Metformin in Individuals With Fragile X Syndrome (Met) (NCT03479476)	250 mg–2000 mg	Phase 2 Phase 3	Metformin may be a potential candidate for targeting multiple intracellular functions in neurons that are impaired in Fragile X Syndrome [151].	Completed
**Cardiovascular Disease**	Metformin and Prevention of Cardiovascular Events in Patients With Acute Myocardial Infarction and Prediabetes (MIMET) (MIMET) (NCT05182970)	500 mg–2000 mg	Phase 3	To be determined	Recruiting
	Carotid Atherosclerosis: MEtformin for Insulin ResistAnce Study (CAMERA) (NCT00723307)	850 mg tablet twice daily	Phase 4	Metformin did not affect carotid intima-media thickness in non-diabetic patients with high risk of cardiovascular disease [152].	Completed
	Efficacy of Metformin as add-on Therapy in Non-Diabetic Heart Failure Patients (NCT05177588)	1000–2000 mg/day	Phase 4	Metformin reduced left ventricular ejection fraction, improved total antioxidant capacity, and prevented the increase in left ventricular mass index compared with standard of care [153].	Completed

* The names of clinical trials included in this table are the same as listed on https://clinicaltrials.gov/ (accessed 3 November 2024).

## Data Availability

No new data were created or analyzed in this study. Data sharing is not applicable to this article.

## Abbreviations

AD: Alzheimer’s Disease, AMI: acute myocardial infarction, AMPK: 5′ adenosine monophosphate (AMP)-activated protein kinase, ApoA5: apolipoprotein A5, ASD: autism spectrum disorders, BBB: blood–brain barrier, BDNF: brain-derived neurotrophic factor, ED: erectile dysfunction, eIF4G: eukaryotic translation initiation factor 4 gamma, EMT: epithelial-to-mesenchymal transition, ERK: extra-cellular signal regulated kinases, ETC: electron transport chain, FASD: fetal alcohol spectrum disorders, FDA: U.S. Food and Drug Administration, FoxO3a: forkhead box O3a, FMR1: fragile X messenger ribonucleoprotein 1, GAD: glutamate decarboxylase, GI: gastrointestinal, Glu: glutamate, HDAC: histone deacetylases, HMGB1: high mobility group box 1, HO-1: heme-oxygenase 1, I/R: ischemia/reperfusion, IL-6: interleukin 6, Il1β: interleukin-1 beta, KATs: histone acetyltransferases, LMK1: liver kinase B1, MATE: multidrug and toxin extrusion, MATE2-K: multidrug and toxin extrusion 2-K, *MECP2* (human gene): methyl CpG-binding protein 2, *Mecp2* (murine gene), MeCP2 (protein), mEPSC: miniature excitatory postsynaptic currents, MFN2: mitofusin 2, mIPCs: miniature inhibitory postsynaptic currents, miRNA: microRNA, MMP-9: matrix metalloproteinase-9, MPTP: N-methyl-4-phenyl-1,2,3,6-tetrahydropyridine, MS: multiple sclerosis, mTORC1: mechanistic target of rapamycin complex, NAFLD: non-alcoholic fatty liver disease, Nrf2: nuclear factor erythroid 2–related factor 2, NSCLC: non-Small cell-lung cancer, OCT: organic cation transporters, OPA1: optic atrophy type 1, OXTR: oxytocin receptor gene, p-eIF4E: phospho-eukaryotic translation initiation factor 4E, p-ERK: phospho-extra-cellular signal regulated kinases, PCOS: polycystic ovary syndrome, PD: Parkinson’s Disease, PDE5: phosphodiesterase 5, PGC-1α: peroxisome proliferator-activated receptor gamma coactivator 1-alpha, PI3K/AKT: phosphatidylinositol 3-kinase/Akt, PMAT: plasma membrane monoamine transporter, PSD95: postsynaptic density protein 95, REDD1: regulated in development and DNA damage responses 1, ROS: reactive oxygen species, RTT: Rett Syndrome, SCD1: stearyl-coenzyme A desaturase 1, Shh: Sonic hedgehog, sEPCs: spontaneous excitatory postsynaptic currents, SNAP25: synaptosomal associated protein 25, T2D: type 2 diabetes mellitus, TGFβ: transforming growth factor beta, TGFβ1: transforming growth factor beta 1, TGFβR2: transforming growth factor beta receptor 2, TNFα: tumor necrosis factor alpha.
